# Informed Switching Strongly Decreases the Prevalence of Antibiotic Resistance in Hospital Wards

**DOI:** 10.1371/journal.pcbi.1001094

**Published:** 2011-03-03

**Authors:** Roger D. Kouyos, Pia Abel zur Wiesch, Sebastian Bonhoeffer

**Affiliations:** Institute of Integrative Biology, ETH Zurich, Zurich, Switzerland; Imperial College London, United Kingdom

## Abstract

Antibiotic resistant nosocomial infections are an important cause of mortality and morbidity in hospitals. Antibiotic cycling has been proposed to contain this spread by a coordinated use of different antibiotics. Theoretical work, however, suggests that often the random deployment of drugs (“mixing”) might be the better strategy. We use an epidemiological model for a single hospital ward in order to assess the performance of cycling strategies which take into account the frequency of antibiotic resistance in the hospital ward. We assume that information on resistance frequencies stems from microbiological tests, which are performed in order to optimize individual therapy. Thus the strategy proposed here represents an optimization at population-level, which comes as a free byproduct of optimizing treatment at the individual level. We find that in most cases such an informed switching strategy outperforms both periodic cycling and mixing, despite the fact that information on the frequency of resistance is derived only from a small sub-population of patients. Furthermore we show that the success of this strategy is essentially a stochastic phenomenon taking advantage of the small population sizes in hospital wards. We find that the performance of an informed switching strategy can be improved substantially if information on resistance tests is integrated over a period of one to two weeks. Finally we argue that our findings are robust against a (moderate) preexistence of doubly resistant strains and against transmission via environmental reservoirs. Overall, our results suggest that switching between different antibiotics might be a valuable strategy in small patient populations, if the switching strategies take the frequencies of resistance alleles into account.

## Author Summary

Infections with bacterial pathogens that are resistant against antibiotics are an important cause of mortality and morbidity in hospitals. One possibility to minimize this burden of antibiotic resistance is to coordinate the use of several drugs at the level of a single hospital ward. Here, we use a computational model of a hospital ward in order to assess the performance of several such strategies that take into account the frequency of antibiotic resistance in the hospital ward. We assume that information on resistance frequencies stems from microbiological tests, which are performed routinely in order to optimize individual therapy. Thus the strategy proposed here represents an optimization at population-level, which comes as a free byproduct of optimizing treatment at the individual level. We find that in most cases our informed strategy can substantially reduce the prevalence of antibiotic resistance. We show that the performance of an informed strategy can be improved substantially if information on resistance tests is integrated over a period of one to two weeks. Overall, our results suggest that switching between different antibiotics might be a valuable strategy in small patient populations, if the switching strategies take the frequencies of resistance alleles into account.

## Introduction

The increasing prevalence of antibiotic resistance in nosocomial infections is a serious threat for clinical care and an important cause for mortality and morbidlity as well as a substantial driver of health care costs [1]. Several strategies to coordinate the use of different drugs and thereby limiting the spread of antibiotic resistance have been proposed. The most prominent such strategies are Cycling (sequential use of different drugs), and Mixing (simultaneous use of different drugs in different patients). The rationale behind cycling is that strains resistant to the formerly used drug may decrease in frequency or even disappear in the off-period. Mixing, on the other hand, creates a strong environmental heterogeneity that makes it difficult for the pathogen to adapt. Concerning these two strategies, the clinical literature is inconclusive [2], [3], while the consensus in most of the theoretical literature is that mixing almost always outperforms cycling [4], [5], [6] (see however also the discussion in [7], [8], [9]). The intuitive explanation for this pattern is that mixing leads to more heterogeneity and hence hinders the adaptation of the bacterial population against the antibiotic agents [4]. Thus it seems that periodic switching of treatment regimes does not help to alleviate the burden of antibiotic resistance in hospitals. On the other hand, treatment decisions that take institution-antibiograms into account [10] may lead to a cycling-like pattern in which antibiotics are withdrawn when resistance rises and re-instituted when resistance becomes more rare. It is often recommended that resistance surveillance should be used as a guideline for empirical therapy (i.e. therapy that is initiated before microbiological results are available) [11]. However, it is difficult to disentangle the effects of this particular strategy from other simultaneously used approaches such as restriction of antibiotic usage [12].

Here, we use an epidemiological model for a hospital ward to show that contrary to the current views switching between different regimes of empirical therapy (i.e. treatment before the causative pathogen and its resistance profile are known) can reduce antibiotic resistance. The switching regime proposed here differs from the traditional ones in [5] and [4] by taking the frequencies of the resistant strain in the hospital into account. Thus in contrast with “blind” periodic switching strategies, we analyze informed switching strategies (ISS) similar to the ones that arise by antibiogram-guided therapy and show that such strategies can serve as valuable strategy to curb resistance.

## Results

**Figure 1:**
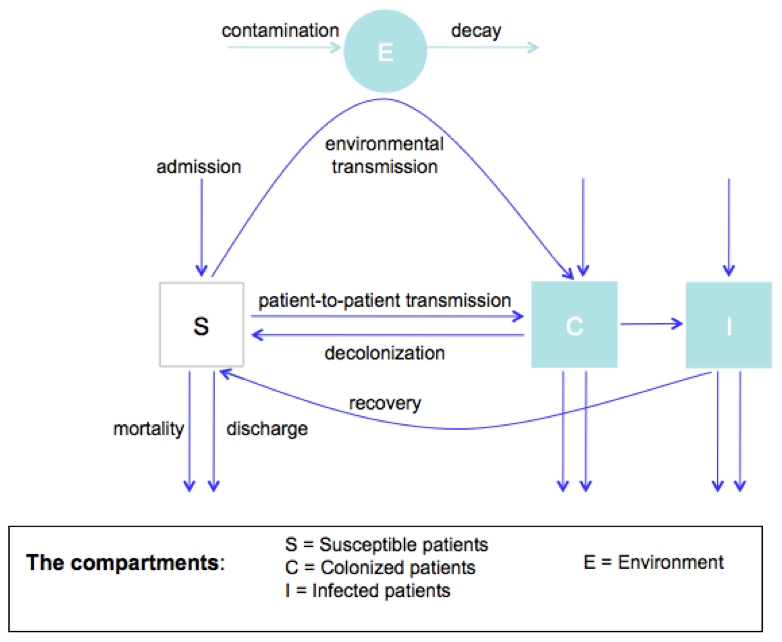
Flow chart of the model.

In this study we use an epidemiological model (see Figure 1 and Tables 1–2) in order to consider the impact of several different informed switching strategies (ISS), which coordinate the use of two broad-spectrum antibiotics A and B on the level of a single hospital ward (see Table 3 for a mathematical characterization of the considered treatment strategies). The common element of these ISS is that if the resistance mutation against one drug is suspected to have gone extinct only this “resistance-free” drug is deployed. In this way, these strategies exploit the high frequency of stochastic extinctions of resistant strains caused by the small population sizes in hospitals. The crucial question for the practical value of an ISS is whether such a strategy can substantially reduce the burden of antibiotic resistance even if it is based on the imperfect information, which can be obtained realistically. Here we model the following realistic scenario of how such information may be obtained: Commonly, symptomatically infected patients are first treated empirically with a broad spectrum antibiotic, then a resistance profile (microbiological tests) is determined (this usually requires 1–2 days), which guides further therapy (optimally with a narrow spectrum antibiotic). In our model we assume that the ISS are based on the information obtained through these microbiological tests, which are made to guide non-empirical therapy of individual patients. Therefore, the model makes the realistic assumptions that i) the information on the resistance status of a symptomatically infected patient is only available after a delay of 2 days on average and ii) that upon the availability of these test-results, the patient is immediately put on a narrow-spectrum therapy regimen against which the pathogen is susceptible. We consider two main classes of ISS: those, which are based on a snapshot of resistance frequencies (i.e. on the frequency of resistance mutations among the infected patients that are currently in the ward and of which microbiological results have been obtained) or those, which integrate information of resistance over a certain time window. Whereas the first class of strategies is simpler to understand from a population biological point of view, we will argue that strategies of the second class are recommendable for clinical practice. We use two measures to assess the success of the ISS (see method section): the prevalence of resistance mutations in the ward and the number of inappropriately treated patients. In fact, it has been shown that inappropriate initial (empirical) treatment increases mortality since severely infected patients might die before treatment can be adjusted [13], [14]. For both measures the success of an alternative strategy is measured relative to mixing: if m_M_ and m_A_ denote the value of the measure for mixing and the alternative therapy, respectively, then the success of the alternative therapy is quantified by Δm = (m_A_−m_M_)/m_M_. Thus, the more negative Δm, the better the strategy.

**Table 1:** Default model parameters of the model.

Parameter	Explanation	Default Value
p_S_	Proportion susceptible among incoming patients	80%
p_c_	Proportion carriers (infected and colonized) among incoming patients	20% [21]
p_i_	Proportion of symptomatically infecteds among incoming patients	5%
p_AB_	Proportion AB-resistant carriers among incoming infecteds and colonized patients	0%
p_A0_	Proportion A-resistant carriers among incoming infecteds and colonized patients	5%
p_0B_	Proportion B-resistant carriers among incoming infecteds and colonized patients	5%
p_00_	Proportion carriers of the completely susceptible strain among incoming infecteds and colonized patients	1-p_AB_-p_0B_-p_A0_
t_R_	Rate with which symptomatically infected patients receive a microbiological test and are switched to a narrow spectrum antibiotic.	0.5 d^−1^ [22]
β	transmission rate (for colonized and infecteds)	0.05 d^−1^ *
a	Rate with which empty beds are filled up	2 d^−1^
c	Rate of treatment cessation in uninfected or asymptomatically infected patients	1/5 d^−1^
T	Observation period over which results are averaged	30*365 d
c_A0_	cost of resistance against A	10% [23]
c_0B_	cost of resistance against B	10% “
c_AB_	cost of resistance against A and B	20% “
r_cl_	Average time until carrier is not infectious when appropriately treated	2 days [24]
*r_p_*	Rate of progression from colonized to infected	1/7 d^−1^
pr	Cycling period	90 d [3]
N	Number of beds	20
l	Discharge + Death rate for asymptomatic patients	1/7 d^−1^ **
l_i_	Discharge + Death rate for symptomatically infected patients	1/21+1/7 d^−1^
f_A0_, f_0B_	Frequency of infected patients treated with drug A or B	see Table 3
*Extension with environmental transmission in the hospital*		
c_E_	Colonization rate of environment	1/10***
l_E_	Turn-over rate of environment	1/30 d^−1^ [25]
β_E_	Transmission rate from environment	β* l_E_/c_E_ ***

*“Colonization pressure”, i.e. the frequency of both asymptomatic and symptomatic carriers in a hospital ward has been shown to be a major risk factor for the acquisition of a nosocomial pathogen [26]. It also has been shown for clostridium difficile, that environmental contamination occurred for both symptomatic and asymptomatic infections [27]. Nevertheless, it is conceivable that e.g. in symptomatically infected patients with diarrhea infectivity is much higher than in asymptomatic patients. However, since the connection between carriage and infection is established and data on potential differences in infectivity between symptomatic and asymptomatic patients are scarce, we chose not to distinguish between these two classes.

**The average length of stay is 8 days in Switzerland (http://www.obsandaten.ch/indikatoren/5_4_1/2005/d/541.pdf, data from 2005) and 5 days in the US (http://www.cdc.gov/mmwr/preview/mmwrhtml/mm5427a6.htm).

***c_E_ is an arbitrary. However, in order to make the environment comparable with direct transmission, the transmission rate β_E_ from the environment is adjusted depending on the decay and colonization rates, such that the R0 remains constant.

**Table 2:** Summary of the different types of events underlying the stochastic implementation of the model.

event class	Description of event	Characterization of event (*E_i_*)	Rate of event (*R_i_*)
1	Admission of uninfected patients	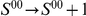	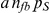
2	Admission of asymptomatically infected patients	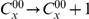	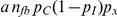
3	Admission of symptomatically infected patients	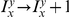	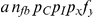 for *y = A0 or y = 0B*
4	Discharge of uninfected patients	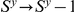	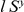
5	Discharge of asymptomatically infected patients	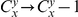	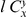
6	Discharge of symptomatically infected patients	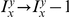	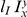
7	Colonization of uninfected patient	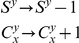	0 if strain *x* is susceptible to treatment *y* 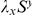 otherwise
8	Progression of untreated asymptomatically infected patients	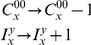	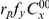 for *y = A0 or y = 0B*
9	Progression of treated asymptomatically infected patients	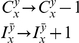	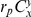 for *y = A0 or y = 0B* ( 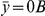 *for* 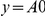 *and* 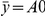 *for* 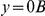 )
10	Microbiological test results become available and patient is put on narrow spectrum treatment	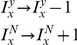	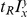 for *y = A0 or y = 0B*
11	Clearance of symptomatically infected patient	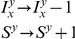	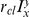 if strain *x* is susceptible to treatment *y* 0 otherwise
12	Cessation of therapy in uninfected patients	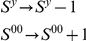	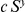 for 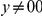
13	Cessation of therapy in asymptomatically infected patients	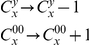	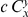 for 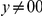

This table follows the description and notation of Gillespie's Direct Algorithm in Box 6.3 of [28]. If not stated otherwise the subscripts *x* range over the four possible strains 00, A0, 0B, and AB and the superscripts *y* over the treatment states 00, A0, 0B and N (thus an event class can contain several events). Furthermore 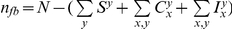 denotes the number of free beds, and 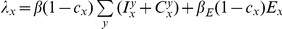 the force of infection for strain x.

**Table 3:** Characterization of deployment strategies.

Strategy	Characterization
mixing	*f_A0_ = *1−*M f_0B_ = *1−*M*
cycling	Alternating between (*f_A0_* = 0, *f_0B_* = 1) and (*f_A0_* = 1, *f_0B_* = 0) with period 90 days
Negative frequency dependent ISS: ISS_--_	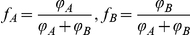
Mixing-like ISS	If *ϕ_A_ = *0 and *ϕ_B_>*0 then *f_A0_* = 1 and *f_0B_* = 0
	If *ϕ_B_ = *0 and *ϕ_A_>*0 then *f_0B_* = 1 and *f_A0_* = 0
	Otherwise *f_A0_* = *M* and *f_0B_* = *M*
ISS_K_	If *t-t_A_*>K and *t-t_B_≤K* then *f_A0_* = 1, *f_0B_* = 0
	If *t-t_B_*>K and *t-t_A_≤K* then *f_0B_* = 1, *f_A0_* = 0
	Otherwise then *f_A0_* = *M*, *f_0B_* = *1−M*
ISS_Last_	If t_A_>t_B_ then *f_A0_* = 1, *f_0B_* = 0
	If t_B_>t_A_ then *f_0B_* = 1, *f_A0_* = 0
	If t_B_ = t_A_ then *f_A0_* = 0.5, *f_0B_* = 0.5

M is a pre-specified constant, characterizing the relative use of drugs A and B in mixing phases (if not stated otherwise *M = 0.5*). 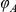 (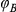) refers to the frequency of the strain resistant to A (B) among patients with a known resistance profile (i.e. patients in treatment class *N*). Thus, 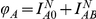 and 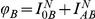. *t* is the current time point *and t*_A_ (*t*_B_) the latest time-point at which resistance mutations against A (B) have been detected. In terms of the model described in Table 2, the time *t*_A_ (*t*_B_) is given by the latest time-point at which an event of class 10 with y = A0 (y = 0B) has occurred.

**Figure 2:**
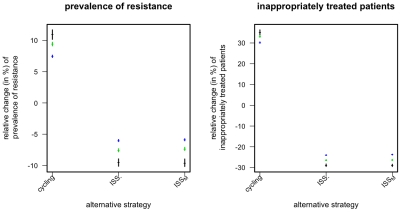
Relative change in prevalence of resistance mutations and of inappropriately treated patients compared to mixing for different snapshot-based alternative strategies. Points correspond to the mean over 10^4^ simulations, error-bars correspond to the 95% confidence interval of the mean, inferred through 1000 bootstrap samples. Color indicates the prevalence of the resistant strains (*p_A0_*+*p_0B_*) among incoming infected and colonized patients (black: 2% green: 10% blue: 20%).

In order to study the population biological basis of the ISS, we start by considering the simpler snapshot-based ISS: The negative-frequency-dependent informed switching, ISS_-_, and the mixing-like informed switching, ISS_M_. Both strategies deploy only one drug if, among the infected patients in the ward whose resistance status is known, there are both no reports of strains resistant against this drug and at least one report of strains resistant against the other drug (see Table 1). If no resistance mutation is present both drugs are used at equal frequencies. The two strategies differ with respect to their deployment of antibiotics when both resistance mutations are present. In this case, ISS_-_ deploys both antibiotics inversely proportional to the momentary frequency of the corresponding resistance mutations, whereas ISS_M_ deploys both antibiotics at equal frequencies. We find that informed switching clearly outperforms both mixing and periodic cycling (Figure 2). By contrast the difference between the two strategies ISS_-_ and ISS_M_ is marginal (Figure 2). Thus the central aspect of the strategies is the coordinated deployment of antibiotics in those phases when one resistance mutation is extinct in the hospital. This fact indicates that the success of ISS is essentially a stochastic phenomenon, as extinctions are chance effects facilitated by small hospital sizes. In accordance with this interpretation and with earlier work[8], [9], we find that, in the deterministic version of our model, the ISS- strategy leads to no substantial improvement over mixing (results not shown, but see section: *effect of population size*).

**Figure 3:**
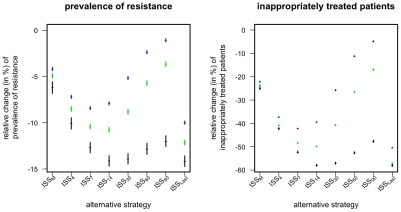
Relative change in prevalence of resistance mutations and of inappropriately treated patients compared to mixing for different ISS that integrate resistance frequencies over time. Points correspond to the mean over 10^4^ simulations, error-bars correspond to the 95% confidence interval of the mean, inferred through 1000 bootstrap samples. Color indicates the prevalence of the resistant strains (*p_A0_*+*p_0B_*) among incoming infected and colonized patients (black: 2% green: 10% blue: 20%).

Considering only the current symptomatically infected patients with a microbiological test leads to an imprecise estimate of the resistance frequencies in the ward, as these patients represent only a small fraction of all carriers. However, as the detection of one infection with a resistant strain is indicative of other such infections (which often persist after the detected case has been cleared) the imprecision can, in part, be compensated by integrating the information over several time points. The strategies ISS_K_ (with K = 4,7,14,30,60,90) integrate information over several time-points in the following way: A resistant strain is considered extinct if no symptomatically infected patient with a microbiological test that detected this strain has been in the hospital for the last K days (see Table 3). Thus ISS_K_ integrates the infection status (of patients with a test) over the last K days. Again, if only one of resistance mutations is considered extinct according to the above criterion, then only the corresponding drug is used. If both or no resistance mutation is considered extinct both drugs are used at equal frequencies. The disadvantage of these ISS_K_ is that they require the choice of the length of the integration time window. An alternative way to integrate information on resistance prevalence over several days without this drawback is the following (ISS_Last_): If at a given time-point the drug resistance-mutations against *A* and *B* have been last detected (among the patients with known resistance status) *t_A_* and *t_B_* days ago, then use that drug for which this time-span is larger. If t_A_ and t_B_ are equal (in particular if both resistance mutations are simultaneously present at the given time point) then both drugs are used at the same frequency. We find that both ways of integrating the information on resistance frequencies can indeed substantially improve the performance of the ISS and that the overall best results can be achieved for ISS_Last_ and for the ISS_K_ with K = 7 or 14 (Figure 3). Accordingly we will focus on these two optimal strategies ISS_7_ and ISS_Last_, when assessing in the following the robustness of our results with respect to several important aspects of the model.

### Impact of different levels of preexistence of the resistant strains

**Figure 4:**
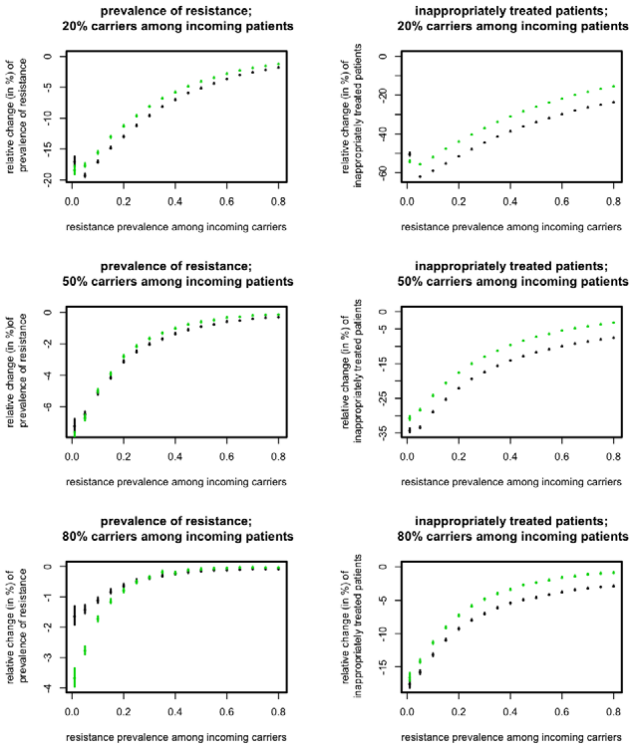
Relative change of resistance prevalence(left column) and inappropriately treated patients (right column) compared to mixing for ISS_7_ (green points) and ISS_Last_ (black points). The figures show the change induced by ISS_7_ and ISS_Last_ as a function of the resistance prevalence among incoming carriers, p_A0_+p_0B_, (x-axes) and for different fractions of carriers, p_C_, among incoming patients (rows). Points correspond to the mean over 10^4^ simulations, error-bars correspond to the 95% confidence interval of the mean, inferred through 1000 bootstrap samples.

One of the major factors determining the success of the ISS is the frequency of resistant strains among incoming patients. The ISS are based on extinctions of the resistance mutations, which is counteracted by the frequent reintroduction of these strains. Accordingly, we find that the success of ISS decreases with increasing influx of resistant strains (Figure 4). However, the advantage of ISS remains substantial unless an unrealistically large fraction of incoming patients carries the resistant strain of the pathogen.

### Impact of different progression rates

**Figure 5:**
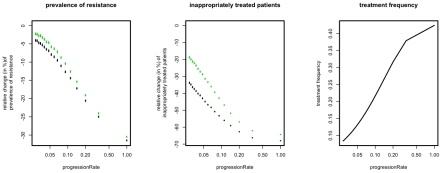
Relative change of resistance prevalence and inappropriately treated patients compared to mixing for ISS_7_ (green points) and ISS_Last_ (black points). The figures show the change induced by ISS_7_ and ISS_Last_ as a function of the rate of progression, *r_P_*, (x-axes). The right panel shows how treatment frequency increases as a function of the *r_P_*. Points correspond to the mean over 10^4^ simulations, error-bars correspond to the 95% confidence interval of the mean, inferred through 1000 bootstrap samples.

The progression rate is important mainly because it affects the fraction of symptomatically infected patients and therefore the frequency at which the antibiotic is used. With increasing progression rate one would expect an increasing use of the antibiotic and hence an increasing impact of the applied strategy. Indeed, we find that both ISS_7_ and ISS_Last_ are especially effective for fast progressing diseases (Figure 5). However, even for moderate and low progression rates, the ISS still confer a substantial advantage in terms of reducing inappropriately treated patients.

### Preexistence of doubly resistant strains

**Figure 6:**
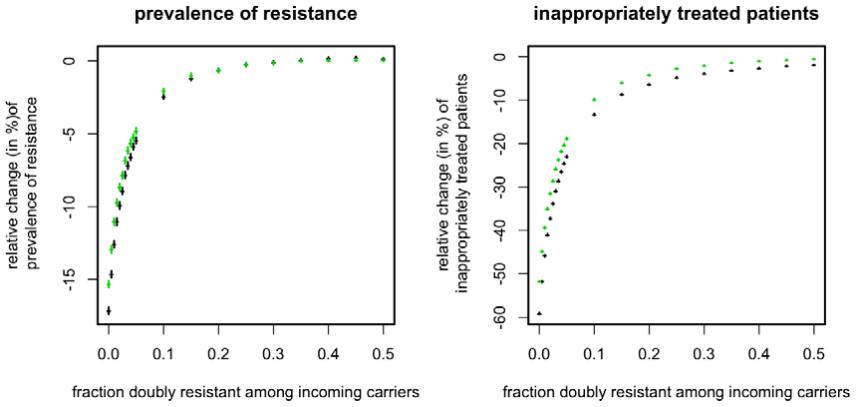
Relative change of resistance prevalence and inappropriately treated patients compared to mixing for ISS_7_ (green points) and ISS_Last_ (black points). The figure shows the change conferred by ISS_7_ and ISS_Last_ as a function of p_AB_, the prevalence of the doubly resistant strain among incoming carriers (x-axes). Points correspond to the mean over 10^4^ simulations, error-bars correspond to the 95% confidence interval of the mean, inferred through 1000 bootstrap samples.

The preexistence of doubly resistant strains among incoming patients has been argued to render treatment strategies futile, i.e. strategies perform equally bad when doubly resistant strains are present [4], [5]. To some extent, this also applies to the ISS discussed above. Specifically we find that the beneficial impact of the ISS decreases substantially as the fraction of incoming patients with a doubly resistant strain increases (Figure 6). The figure, however, also shows that the ISS can still substantially reduce the prevalence of drug resistance even if the frequency of the doubly resistant strain is as high as 5% among colonized and infected patients.

### Transmission via an environmental reservoir

**Figure 7:**
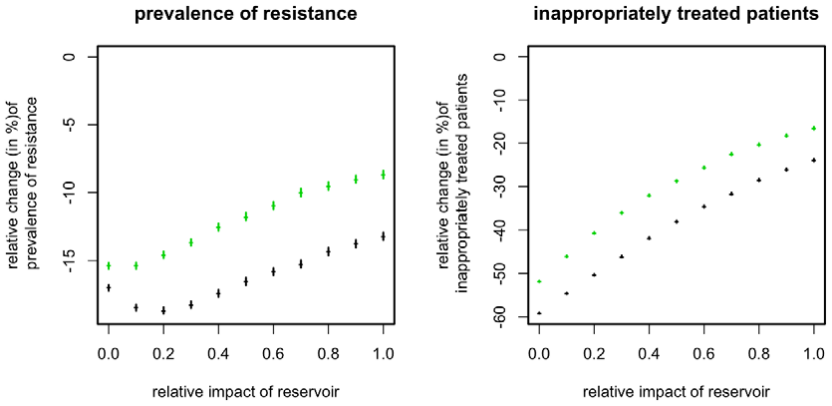
Relative change of resistance prevalence and inappropriately treated patients compared to mixing for ISS_7_ (green points) and ISS_Last_ (black points). The figure shows the change conferred by ISS_7_ and ISS_Last_ for different relative impacts of the environmental reservoir (x-axis). The relative impact of the reservoir is measured as the relative fraction of the force of infection that is mediated via the reservoir. Points correspond to the mean over 10^4^ simulations, error-bars correspond to the 95% confidence interval of the mean, inferred through 1000 bootstrap samples.

As the success of the ISS is essentially a stochastic effect, one would expect that it becomes weaker in the presence of an environmental reservoir. This is because an environmental reservoir exhibits a slower turnover of strains (see Table 1) and hence reduces the extinction risk of resistant strains, i.e. the reservoir can act as a “seedbank” for resistant strains. Indeed, we find that the ISS perform slightly worse in the presence of such a reservoir (Figure 7). However this decrease in the strategies' efficiency is very weak and the improvement achieved by applying ISS_7_ remains substantial even if transmission is uniquely mediated by an environmental reservoir. This indicates that even if the turnover rate of strains in the ward is reduced to that of the reservoir (here: 1/(30 days)) stochastic effects are strong enough to ensure the efficiency of the ISS.

### Impact of population size

**Figure 8:**
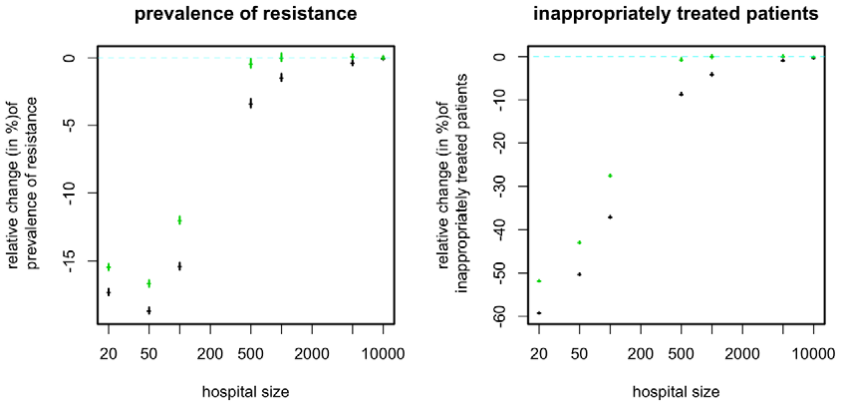
Relative change of resistance prevalence and inappropriately treated patients compared to mixing for ISS_7_ (green points) and ISS_Last_ (black points). The figure shows the change conferred by ISS_7_ and ISS_Last_ as a function of *N*, the number of beds in the ward. In order to keep the R_0_ constant across different population sizes the transmission rate is assumed to be inversely proportional to the number of beds (i.e. β∼1/N). Points correspond to the mean over 10^4^/(N/20) simulations (the number of samples was chosen inversely proportional to the population size because, with the Gillespie algorithm used, simulation time increases proportionally with population size whereas the level of stochastic variation decreases with population size), error-bars correspond to the 95% confidence interval of the mean, inferred through 1000 bootstrap samples.

The way in which the benefit conferred by the ISS depends on population size confirms the stochastic nature of this effect: As expected the benefit essentially disappears for very large population sizes when stochastic effects are expected to be small (Figure 8). Regarding the fraction of inappropriately treated patients the magnitude of the benefit decreases monotonically with increasing population size. Regarding the prevalence of resistance, we observe however a slight increase in the magnitude when increasing the population from 20 to 50. Although it is not entirely clear what causes this increase, it might be that for very small population sizes the subpopulation of patients with microbiological tests gives a very inaccurate picture of the resistance prevalence even if integrated over time. An alternative explanation is that in very small populations, extinction events impede resistance emergence, such that resistance is infrequent regardless of the treatment regimen. In this case, the additional reduction of resistance may be small. However, as soon as the population size exceeds 100 the magnitude of the effect decreases strongly (with regard to both measures) and becomes negligible at population sizes of 500 and beyond.

### Asymmetrical scenarios

**Figure 9:**
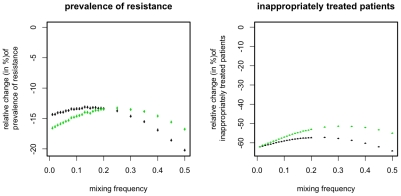
Relative change of resistance prevalence and inappropriately treated patients compared to mixing for ISS_7_ (green points) and ISS_Last_ (black points). The figure shows the change conferred by ISS_7_ and ISS_Last_ for an asymmetric scenario in which p_A0_ = 0.1*2/3, p_0B_ = 0.1*1/3, c_A0_ = 0.2 *1/3 and c_0B_ = 0.2*1/3 (i.e. the less costly mutant is more abundant). The x-axis corresponds to the mixing frequency M (see Table 3). Note that the mixing and ISS_7_ depend on M, whereas ISS_Last_ is independent of M. Points correspond to the mean over 10^4^ simulations, error-bars correspond to the 95% confidence interval of the mean, inferred through 1000 bootstrap samples.

For simplicity, we assumed so far that both resistance genes are symmetrical, i.e. that resistance-costs and prevalence among incoming patients carrying the resistant strain are identical for drug A and B. If this assumption is relaxed, the optimal mixing strategy does not deploy the two drugs at equal frequencies but gives preference to the drug whose resistance mutation is more frequent among incoming patients and less costly. We make the realistic assumption that these two properties coincide: i.e. the less costly mutations are more frequent. We find that in such an asymmetric scenario, the ISS still considerably outperform even the optimal mixing strategy (Figure 9). Interestingly, this scenario provides the only example (apart from a very narrow parameter region in Figure 3) in which the ISS_7_ strategy can outperform the ISS_Last_ strategy. However, the difference is rather small and depends sensitively on choosing the correct mixing strategy for those phases in which the ISS_7_ deploys both drugs simultaneously (see definition of ISS in Table 3).

## Discussion

Previous theoretical studies suggest that from the point of view of preventing the spread of resistance mutations, mixing strategies perform at least as good as strategies that switch between different antibiotics [4], [5]. However, selecting treatment based on cumulative ward antibiograms has been shown to increase adequate therapy [15]. Here, we have shown that such a strategy does not only benefit the single patients receiving appropriate therapy, but may also be used to counteract the spread of resistance. In the highly stochastic setting of small hospital wards, mixing (random treatment) can be substantially outperformed by informed switching strategies, which take the frequency of antibiotic resistance mutations into account. Factors that promote the success of such ISS include the absence of multiply resistant strains and a low frequency of singly resistant strains among the incoming patients. However, even if these conditions are not fulfilled, ISS can still substantially alleviate the burden of drug resistance. Moreover, we found that the magnitude of the effect of ISS decreases with increasing fitness cost, remains however substantial even for large fitness costs (results not shown). Given that, especially in the long term, resistance carries very small costs if any [16], the default fitness cost chosen here (*s* = 0.1) can be considered as an upper bound yielding thus a conservative assessment of the effect of ISS.

In our view, the most promising version of an informed switching strategy is ISS_Last_. Apart from the fact that this strategy outperformed the other versions in almost every setting, it has the advantage that its implementation would be relatively simple: Essentially, it would only require that the dates at which resistant strains have been detected in a given ward are recorded, and that for every new patient that drug is used for which the last isolation date is most distant. The main case in which we found this strategy not to be the best choice, was if among incoming patients resistance mutations against one drug was much more common than resistance mutations against the alternative drug. In this situation ISS_Last_ clearly outperformed mixing. However, it was slightly worse than the following alternative strategy: Always using the antibiotic less common among incoming patients, except if a resistance mutation against this drug has been detected in the past seven days, in which case only the alternative drug has to be deployed (formally this corresponds to ISS_7_ with *M = *0, see Table 3). However the additional improvement conferred by this strategy was modest and does in our view not outweigh the larger simplicity and robustness (independence of an integration time-window) of ISS_Last_.

The benefit conferred by the ISS is a result of the underlying stochasticity of resistance prevalence in the hospital. This is demonstrated by our finding that the magnitude of the effect becomes negligibly small as soon as the population size is above 500–1000. This is consistent with the findings of [8], [9] which found in a deterministic model no (or no substantial) improvement is conferred by an “adaptive” strategy similar to the ISS used here. The fact that ISS are very effective for small population sizes but have limited success at large population sizes, suggests that switching strategies should be implemented at the ward level rather than at the hospital level, especially in large hospitals.

The information underlying the switching strategies considered here is a byproduct of microbiological resistance tests, which are usually done in clinical practice in order to optimize individual treatments. While the recent HICPAC guidelines, “Management of Multidrug-Resistant Organisms in Healthcare Settings,” [11] recommend at least annual updates, the strategies proposed here are not based on discrete updates. Instead the information acquired from microbiological tests would have to be integrated into the decisions as it is generated in the course of optimizing individual treatments. Also, in accordance with current recommendations [17], [18] we follow resistance in one single hospital ward, not in the whole institution, such that the cumulative antibiogram of the respective wards should be chosen for informed switching. As the success of an informed switching strategy depends on the quality of the information on the frequencies of resistance genes, the success of the strategy can be further improved by sampling also asymptomatically infected patients (results not shown). However, we think that the strategies we have proposed in this study represent the most realistic option, given that they come at no additional cost other than compiling the available data from microbiological tests. Furthermore, the inclusion of isolates from asymptomatic patients is not recommended by the Clinical and Laboratory Standards Institute [CLSI] [19]. Thus although the extent of information is important for informed switching strategies, a realistic and often available degree of knowledge seems to be sufficient for a successful implementation of the strategy.

In summary, we have shown that coordinated informed switching of the antibiotic deployed in a hospital ward can outperform mixing as a strategy to limit the spread of antibiotic resistance of nosocomial pathogens. This theoretical result is especially interesting, since the impact of surveillance-guided therapy is often difficult to assess [12].

## Methods

We consider a compartmental epidemiological model that describes a single hospital ward. We further consider two empirical broad spectrum antibiotics, to which we refer as drug A and B. Accordingly, we follow four genotypes: wild type (sensitive to both drugs), resistant against A and sensitive to B, resistant against B and sensitive to A, and resistant against both drugs. Patients are classified as being susceptible (S), colonized (C; i.e. asymptomatic carriers), or infected (I; i.e. symptomatic carriers). Furthermore, the compartments are subdivided according to the treatment status and (for I & C) according to the genotype of the carried strain. In addition, we follow a pathogen reservoir outside the patients (E), which describes environmental contamination but may also describe the dynamics resulting from the transient colonization of health care workers; although these are not modeled explicitly. Finally, we assume that symptomatically infected patients undergo a microbial test (with a rate *t_R_*) after which they are switched to an appropriate narrow spectrum antibiotic for which we assume that resistance is negligible. These test-results provide the information on resistance frequencies upon which the ISS are based.

Figure 1 summarizes the population dynamics of the model for a single strain and Table 1 lists the parameters and their default values (which are used if not declared explicitly otherwise). We used parameter values from clinical literature as far as they are available.

We assume a fixed number of 20 beds in the hospital ward. As soon as a bed is free, patients of all classes carrying pathogens of all genotypes may be admitted at frequencies that are assumed to be constant over the observed timeframe (see Table 1). The proportion of incoming patients belonging to the three main compartments S, C and I is determined by the parameters *p_S_, p_C_* and *p_I_* (see Table 1). The proportion of patients carrying the genotypes wt, A, B, and AB is given by the parameters p_AB_, p_A0_ and p_0B_. Upon admission patients are not treated unless they are symptomatic carriers (i.e. we focus on non-prophylactic treatment). Upon transition to the “infected” compartment all patients are treated with a broad-spectrum antibiotic according to the current treatment strategy (Mixing, Cycling or informed switching). After clearance of the pathogen, treatment is ceased at a rate of 1/5 d^−1^.

### Detailed model description

Here we consider a stochastic version of the model described above. Specifically, the state of the patient-population in the ward is given by the discrete variables 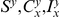 referring to the number of susceptible (S) asymptomatically infected (C) and symptomatically infected (I) patients with treatment status y and infection status x. The infection status can be either “infected with the strain susceptible against both drugs” (x = 00), “infected with the strain susceptible against A but resistant against B” (x = 0B), “infected with the strain susceptible against B but resistant against A” (x = A0), or “infected with the strain resistant against both drugs” (x = AB). The treatment status can be either “treatment with no drug” (y = 00), “treatment with drug A” (y = A0), “treatment with drug B” (y = 0B), or “treatment with a narrow spectrum antibiotic” (y = N). We assume that the narrow-spectrum antibiotic is only administered after microbiological tests and that hence the infection status of patients in this treatment class is known. The state of the environmental colonization is given by the density E_x_ of the strain x in the environment.

The patient population is simulated stochastically according to Gillespie's Direct Algorithm[20]. The full characterization of this model is given by Table 2, which lists the different events (and rates) that constitute the model. The following points should be noted concerning these events:

- Symptomatically infected patients are always subject to monotherapy, thus 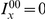 for all *x* and accordingly the dynamics of these variables is not considered.
- According to the model dynamics, asymptomatically infected patients are never treated with narrow spectrum antibiotic hence the variables 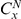
are constantly 0 and therefore ignored in the model.
- Upon admission or progression, symptomatically infected patients are first treated with a broad-spectrum antibiotic (empirical therapy) (see event classes 3,8, and 9 in Table 2). Then these patients receive microbiological tests with a rate *t_R_* and are switched to a narrow spectrum-treatment as soon as these test results are available (event class 10).
- If an asymptomatically infected patient becomes symptomatic despite treatment with a broad-spectrums antibiotic, then the broad-spectrum drug used for that patient is switched (from A to B and from B to A)(event class 9).
- The rates for several event-classes (classes 7 and 11) depend on whether strain x is sensitive to treatment y. Strain 00 is susceptible against all drugs. Strain A0 is susceptible against drug B and against the narrow-spectrum antibiotics (N). Strain 0B is susceptible against drug A and against the narrow-spectrum antibiotics (N). Strain AB is only susceptible against narrow-spectrum antibiotics (N).
- The broad spectrum antibiotics are A and B are used at frequencies f_A0_ and f_0B_. These frequencies are determined by the treatment strategy deployed. A summary of treatment strategies is given in Table 3.

As the dynamics of the environmental compartment is not directly affected by the fluctuations of the patient population, the variables *E_x_* describing the environmental reservoir are updated according to the ODE system
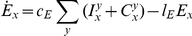



The success of the treatment strategies (summarized in Table 3) is measured by their impact on the prevalence of resistance given by 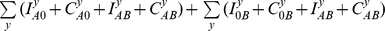


 (note that double resistant strains are counted twice) and by their impact on the number of inappropriately treated patients given by 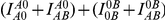. (Note that for this measure we take only symptomatically infected patients into account, because it is in that group that inappropriate treatment will have the most severe clinical consequences).
